# Using *personal health records* for medication continuity during transition of care: An observational study

**DOI:** 10.1177/18333583241270215

**Published:** 2024-08-26

**Authors:** Martina Francis, Peter Francis, Meredith Makeham, Melissa T Baysari, Asad E Patanwala, Jonathan Penm

**Affiliations:** 1 Department of Pharmacy, Royal Prince Alfred Hospital, Camperdown, New South Wales, Australia; 2 Faculty of Medicine and Health, School of Pharmacy, University of Sydney, Sydney, New South Wales, Australia; 3 Department of Infectious Diseases, Blacktown Hospital, Blacktown, New South Wales, Australia; 4 Department of Pharmacy, Prince of Wales Hospital, Randwick, New South Wales, Australia; 5 University of Sydney, Faculty of Medicine and Health, School of Medical Sciences, Sydney, New South Wales, Australia

**Keywords:** e-health, electronic health records, hospital records, medication errors, medication reconciliation, patient transfer, personal health records

## Abstract

**Background:** National Personal Health Records (PHRs) have been proposed to improve the transfer of medication-related information during transition of care. **Objective:** To evaluate the concordance between the medications captured in the Australian national PHR, My Health Record (MyHR), and the pharmacist obtained best possible medication history (BPMH) for patients upon hospital admission. **Method:** This prospective observational study used a convenience sample of hospital patients. For newly admitted patients, the investigating pharmacist obtained a BPMH and then compared it to the medication list captured in MyHR. Upon comparison, the medications were categorised into either complete match, partial match or mismatch. Medications with a complete or partial match were grouped together. Medications with deviations were then assessed for risk based on their potential consequence, and reported descriptively. A multivariable logistic regression was conducted to assess the factors associated with a drug being mismatched. **Results:** A total of 82 patients were recruited, with a cumulative total of 1,207 medications documented. Of the 1,207 medications, 714 (59.2%) medications were documented as a complete/partial match. The remaining 493 (40.8%) medications were mismatched. Of the 493 mismatched medications, 442 (89.7%) were deemed low-risk deviations and 51 (10.3%) were deemed high-risk. A medication was more likely to be mismatched, rather than completely/partially matched, if it was a regular non-prescription medication, or “when-required” prescription medication, or “when required” non-prescription medication, or if it was administered parenterally. **Conclusion:** National PHRs may be a secondary source to either confirm a patient’s medication history or be used as a starting point for a BPMH.

## Introduction

Transition of care has been identified as a key area at risk of medication errors (National Transitions of Care Coalition, 2008; Russell et al., 2013). The World Health Organization (WHO) reported that up to 97% of adult patients experience at least one medication discrepancy on admission (WHO, 2019). The WHO has also reported this to be a global priority in the Patient Safety Action Plan for 2021–2030, as it has remained a consistent issue. These medication discrepancies are due to various reasons when a patient moves between hospital and primary care, including but not limited to: patients being unable to recall all their medications (due to potential baseline cognitive issues, complex medical regimens, acute exacerbation of disease state or other factors); patient/carer poor health literacy; or incomplete/inaccurate information transferred from the primary care setting (WHO, 2019) to the acute setting (e.g., the emergency department); or there is no communication tool that links all medication-related information across settings (National Transitions of Care Coalition, 2008). These factors contribute to a lack of medication continuity upon transition of care, such as hospital admission.

One strategy to improve medication continuity upon hospital admission is medication reconciliation (WHO, 2019). The purpose of medication reconciliation is to ensure that the medications that should be prescribed match those that are prescribed following a transition of care (National Transitions of Care Coalition, 2008; WHO 2019). The process of medication reconciliation involves first obtaining a medication history, and then verifying it with a secondary source (such as community pharmacy dispensing records or General Practitioners (GPs)) to ensure it is a best possible medication history (BPMH). Finally, the BPMH is compared to the inpatient medication list and any identified unintended deviations are appropriately rectified (Francis et al., 2022). The process of conducting medication reconciliation takes an average time of approximately 30 min (Gleason et al., 2012), but can take up to approximately 92 min in complex patients, with the majority of the time attributed to obtaining, verifying and documenting the primary BPMH (Meguerditchian et al., 2013).

Due to the time-consuming nature of obtaining, verifying and documenting BPMHs (Francis et al., 2022) a potential source that may support obtaining BPMHs in a time-efficient manner is national personal health records (PHRs). PHRs, which are a subset of electronic health records (EHRs), are computer-based platforms that digitise health information such as medications, and can act as a communicative tool between transition of care settings (e.g., between primary care and acute setting) (National Transitions of Care Coalition, 2008; Russell et al., 2013; WHO, 2019). The difference between EHRs and PHRs is that PHRs allow health consumers to access and manage their health information and provide access to their healthcare providers and other carers who need it (Markle Foundation Connecting for Health, 2003).

National PHRs have the potential to assist with medication continuity upon hospital admission as they can auto-populate with medication lists from healthcare providers’ prescribing data and community pharmacies’ dispensing data in real-time (National Transitions of Care Coalition, 2008). Furthermore, in PHRs, allow patients to take an active role in their healthcare, which further facilitates medication continuity during transition of care (National Transitions of Care Coalition, 2008; Russell et al., 2013; WHO, 2019). Current literature explores both the potential and the concerns of PHRs. The literature that promotes the potential use of PHRs uses qualitative methods, such as validated questionnaires, surveys and semi-structured interviews to gauge the perspective of different healthcare professionals on the use of PHRs (Al Meslamani et al., 2023; Mullins et al., 2021). While the study by Al Meslamani et al., reported that the majority of pharmacists (71.6%) *agreed* and *strongly agreed* that PHRs will improve the pharmacist’s ability to improve medication review, more than half (55.2%) were concerned about the accuracy of the information within PHRs, and more than one-fifth (20.9%) were concerned about the value of PHRs in pharmacy practice (Al Meslamani et al., 2023). Additionally, the study by Mullins et al. also evaluated the perception of hospital physicians and pharmacists on the national Australian PHR, *My Health Record* (MyHR) (Mullins et al., 2021). The study reported that physicians raised the efficiency-related benefits more frequently and more confidently than pharmacists. Physicians would rely on PHRs as a source of information after-hours and report that the primary use of it, would replace other sources of information, while pharmacists report using the PHR as an additional, secondary source of information (Mullins et al., 2021). Similar to Al Meslamani et al., this study evaluated the perception of utility by healthcare professionals and did not examine the data within the PHR.

Although the results from the aforementioned qualitative literature supports the potential utility of PHRs, the medication information in PHRs requires assessment. Furthermore, there remain certain risks and concerns to implementing PHRs. The Personal Health Workgroup, which represents a public–private collaborative programme of innovators and consumers, reports users mostly fear a breach in security and/or privacy (Markle Foundation Connecting for Health, 2003). To overcome the embarrassment or discrimination if an unauthorised person views their health information, the PHRs system will need to authenticate users and allow them to specify what parts of their PHR will be shared with specific providers and institutions (Markle Foundation Connecting for Health, 2003).

The national Australian PHR, MyHR addresses certain elements of the aforementioned literature. MyHR addresses the concerns regarding security and privacy for patients, as it requires patients to authenticate themselves prior to access and allows them to exercise control as to who views what health information. Although it has the potential capacity to transfer medication-related information upon hospital admission, the data in MyHR has not yet been explored in its ability to support obtaining a BPMH (Russell et al., 2013; WHO, 2019). Therefore, the primary objective of this study was to evaluate the concordance between the medications captured in the Australian national PHR, *MyHR*, and the medications captured in the pharmacist-obtained BPMH for patients upon hospital admission, and thereby evaluate its ability to be a secondary source. The secondary objective was to compare these two medication lists and determine the category, type and severity of any deviations; and identify risk factors for medication deviations.

## Method

### Study design and setting

This was a prospective observational study from 28 September to 18 November 2021, across two large tertiary teaching hospitals with approximately 900 and 750 beds, on predominantly medical wards, in New South Wales, Australia. Ethics approval was obtained from Sydney Local Health District Ethics Review Committee (2019/ETH07525).

### Participants

Patients taking five or more prescription medications who had not opted-out of their national PHR were included in this study. Patients were screened from a convenience sample based on the availability of the pharmacist. This convenience sample is based on the normal workflow of the pharmacist, whereby high-risk patients are prioritised. Patients were excluded if an in-person BPMH could not be obtained. These was primarily patients who had an altered mental or cognitive status (e.g. comatose patient) and no available carer to provide information; or the patient/carer primarily responsible for the medications could not speak English. Furthermore, patients from a community facility (e.g. nursing home) or transferred from a different healthcare facility (e.g. transfer from another hospital) were excluded because their medications are managed by healthcare professionals, and the medication chart brought in by these patients upon hospital admission is predominantly the single source used to obtain a BPMH, and their MyHR is typically not checked.

### Personal health record system

In July 2012, an optional nationwide personally controlled electronic health record (PCEHR) was launched for Australians as a means to allow the secure sharing of health information among an individual’s healthcare providers, while enabling individuals to control their records (Makeham, 2019). However, in July 2018, the PCEHR opt-in model changed and became *MyHR*, whereby all people had a digital health profile provided by the Australian Digital Health Agency (an Australian government statutory agency responsible for electronic health programmes) unless they chose not to have one. The shift to an opt-out model resulted in a large number of Australians now having MyHR, from 5.89 to 23.5 million (The Australian Digital Health Agency, 2020), meaning approximately 90% of Australians have a MyHR (Makeham, 2021). *MyHR* is a national PHR that auto-populates with health information, including, but not limited to, community pharmacy’s dispensing history, shared health summaries from GPs, discharge summaries from hospitals, Australian Immunisation Registry information, pathology, and medical imaging results from connected healthcare providers. Between May 2022–2023, there was a 48% increase in medicine-related documents uploaded to MyHR and a 54% increase in the dispense records uploaded (The Australian Digital Health Agency, 2023). Overall, 95% of public hospitals use MyHR (The Australian Digital Health Agency, 2023), which highlights the potential use of MyHR as a secondary source to support medication continuity upon hospital admission (Makeham, 2021).

The medicines information in the MyHR system is ultimately consumer-controlled. Consumers can express to their HealthCare providers their preference to allow for prescription and dispensing information to flow to MyHR. Furthermore, consumers can also contribute their own medicines information. Therefore, the medicines information in MyHR will ultimately vary between consumers depending on their preferences. Healthcare organisations that have a unique Healthcare Provider Identifier-Organisation (HPI-O) number can securely connect to MyHR and view all medicines information held in a consumer’s MyHR. This view is called the “Medicines View.” The interface used by hospital pharmacists in this study, *HealtheNet*, offers hospital clinicians the “Medicines View,” which was used in our study to capture MyHR medicines information (Makeham, 2019).

### Data collection

The investigating pharmacist obtained a BPMH, without using the patient’s MyHR, and then compared it to the patient’s medication list found in their MyHR. This was completed at the start of their admission to ensure no medication changes or new information was uploaded, and it was a real-time indication of the patient’s medication history upon hospital admission. Any, and all, medication information that was uploaded from the date of hospital admission was disregarded. The patient would remain included, but information from the date of admission was excluded.

All medications listed in the national PHR in the last 6 months were reviewed for inclusion, with the following medications excluded:

(1) Medications categorised as “when required” (both prescription and non-prescription) that had no history (either from pharmacy dispensing records or shared medical health summaries) for more than 3 months, as these were assumed to not be current.
(2) Medications that were only required for a short-period or defined duration, such as antibiotics or post-operative eye drops (assuming their usage was completed prior to hospital admission).
(3) An older medication, when medications were duplicated from the same drug class (e.g. if a patient had metformin and metformin/sitagliptin combination listed in their PHR), because it was assumed that there was a therapy change.
(4) Medications that were listed as “Prescription” in MyHR, with a future date.

### Definitions

On comparing the BPMH and national PHR, a medication deviation was defined as any difference between the pharmacist-obtained BPMH and the medication list in the national PHR. The medication deviations definition was modified from that of a previous Australian study (i.e. “any differences between the student-obtained and pharmacist-obtained BPMH” (Francis et al., 2022). These deviations were initially categorised according to type, as per the medication discrepancy taxonomy (MedTax) (Almanasreh et al., 2020), and then assessed for risk based on their potential consequence, as either insignificant, minor, moderate, major or catastrophic according to risk classification published by The Society of Hospital Pharmacists of Australia (SHPA) (2013). This risk classification assesses the potential risk of harm had no intervention occurred during hospital admission. See Box 1 for definitions of “deviation type” and “deviation consequence.”

**Box 1.** Definitions for deviation type and consequence.

Medication deviation type (Almanasreh et al., 2020)	
Mismatch	Omission, commission, duplication, therapeutic class substitution, allergy or other*
Partial match	Incorrect drug name, strength, frequency, number of dosage units, total daily dose, dosage form, administration route, time of drug administration, duration or therapy length
Medication deviation consequence (The Society of Hospital Pharmacists of Australia, 2013)	
Insignificant	No harm or injuries, low financial loss
Minor	minor injuries, minor treatment required, no increased length of stay or re-admission, minor financial loss
Moderate	Major temporary injury, increased length of stay or re-admission, cancellation or delay in planned treatment/procedure. Potential for financial loss
Major	Major permanent injury, increased length of stay or re-admission, morbidity at discharge, potential for significant financial loss
Catastrophic	Death, large financial loss and/or threat to good will/good name

* Other: Medications where either the prescriber’s intentions are unclear, the patient is non-compliant with the medication, or the medication has variable-dosing, such as warfarin, and is dispensed under multiple strengths.

Medications, with either a complete match or a “partial match,” were classified as “Complete/partial match.” A complete match was defined as the correct drug name, strength and regimen, and a partial match was defined as the correct drug name only. The medications that were omissions (i.e. not captured in the MyHR, but correctly documented in the pharmacist-obtained BPMH) or commissions (i.e. incorrectly captured in the MyHR as a current medication) were classified as “Mismatch.” The other mismatch medication deviation types (drug duplication, therapeutic class substitution or allergy) were filtered out as a result of the exclusion criteria. Team consensus deemed that medications with a complete match or a partial match were categorised together because having a medication listed in the national PHR is sufficient to prompt a healthcare professional to question whether the drug is current and its dosage. This is based on pharmacists being the healthcare professionals primarily responsible for obtaining BPMHs, and as confirmed in literature, pharmacists do not rely on PHRs as a primary or sole source of information (Mullins et al., 2021). Consequently, the decision to categorise medications with a complete match or a partial match together was based on the pharmacists using PHRs as a prompt or secondary source, rather than a replacement or primary source of information to obtain a BPMH. In comparison, a mismatched medication poses a greater risk to medication continuity and subsequent safety. The drug mismatches (omission and commission) were then tabulated against medication type (prescription and non-prescription) to evaluate which medication type predominantly accounted for the different drug mismatch deviations. The drug mismatches with a potential consequence of “insignificant” or “minor” were deemed not-clinically relevant and those deemed “moderate,” “major” or “catastrophic” were deemed clinically relevant. The type and consequence for each deviation was independently categorised by one hospital pharmacist and one hospital medical officer. Any disagreements were discussed with a third senior academic clinical pharmacist and final decisions were decided by 2/3 consensus.

### Variables

The patient variables collected were age, gender, hospital site, admitting clinical specialty, type of admission (planned or emergency) and Charlson Comorbidity Index (CCI) score.

The medication-related variables collected were the type, category and number of medications identified. The type was defined as either regular prescription, regular non-prescription (this included over-the-counter medicines and vitamins taken on a regular schedule), “when required” prescription, and “when required” non-prescription; the category was defined according to the Anatomical Therapeutic Chemical (ATC) classification at the first and second level (WHO Collaborating Centre for Drug Statistics Methodology, 2019); and the medicines were all counted and recorded by individual generic drug (e.g. the combination tablet of irbesartan and hydrochlorothiazide counted as two medicines, not one).

### Sample size

This was an observational study and used a convenience sample from a previous internal quality improvement project where all screened patients were included.

### Statistical analysis

Descriptive statistics were used to report all quantitative variables. For continuous normally distributed data, mean (standard deviation) is reported, and for categorical data, percentages were reported. The primary outcome measure was the proportion of the medications that MyHR captures from the patients’ BPMH with either a complete or a partial match. Therefore, this was descriptively reported as an overall percentage of the total medications documented by the BPMHs. All patient data were complete; hence, there were no missing data in this study. To address our secondary objective, all medication deviations were classified according to ATC categories, MedTax (drug mismatch (omission or commission), and partial match) deviation type and the severity of the deviation discrepancy. These were also reported descriptively.

A multivariable logistic regression was conducted to examine any association between the dependent variable, whether a medicine was mismatched (i.e. omitted or committed), and the following independent variables: medication type (regular prescription, regular non-prescription, when-required prescription and when-required non-prescription); route (oral, parenteral, others) and the ATC classification at the first level only. The dependent variables were chosen as reference due to their frequent usage. However, this does not imply that no mismatches occurred in these categories. For all analyses, a p-value of less than 0.05 was considered statistically significant. Statistical analyses were computed using SPSS (version 28, 2021, IBM Corp, Chicago, IL, USA).

## Results

### Study cohort

From the 91 patients screened, 82 (90.1%) had not opted out of their national EHR. For the included patients, the mean age was 77.3 (SD = 14.7) years and 51.2% were female. The majority of patients were admitted through the emergency department (89.0%) and admissions were not surgery-related (76.8%). As an indication of medical-history complexity, the mean CCI score was 6.6 (SD = 3.0). A similar number of patients were included from each site (54.9% and 45.1%). See Table 1 for patient demographics.

**Table 1.** Patient demographic details.

Variable	Total, *n* = 82
Age, mean (STD)	77.3 (14.7)
Female (%)	42 (51.2)
Site A (%)	45 (54.9)
Emergency (%)	73 (89.0)
Non-surgical admission (%)	63 (76.8)
Charlson comorbidity index score, mean (STD)	6.6 (3.0)

*Note*. STD = Standard deviation.

There was a total of 1,404 medicines documented across both the MyHR and BPMH. Upon reviewing, there were 86% (*n* = 1,207/1,404) that met the inclusion criteria. See Table 2 for details of the included medications. The 14% (*n* = 197/1,404) that were excluded were due to short-term medicines (60%, 119/197); therapy duplication/substitution (18%, 36/197); “when-required” prescription medicines dispensed more than 3 months ago (9%, 18/197); “when-required” non-prescription medicines dispensed more than 3 months ago (6%, 11/197); regular prescribed medicines dispensed more than 3 months ago (2%, 4/197); medicines that were listed as “Prescribed” with a future date (3%, 5/197) and medicines ceased according to a discharge summary (2%, 4/197) (results not tabulated). On average, patients reported taking 15 (SD = 6) home medications upon admission. This represented an average of 9 (SD = 4) regular prescription medicines; 3 (SD = 2) regular non-prescription medicines; 1 (SD = 1) when-required prescription medicine and 2 (SD = 1) when-required non-prescription medicines.

**Table 2.** Details of included medications (*n* = 1,207/1,404).

Total medications, n (mean)	1,207 (14.7)
Regular prescription	702 (58.2)
Regular non-prescription	280 (23.2)
“When required” prescription	72 (6.0)
“When required” non-prescription	153 (12.7)
Deviation type, n (%)	
Complete match	460 (38.1)
Partial match	254 (21.0)
Mismatch	493 (40.8)

### Primary objective – Medication deviations

Of 82 included patients, 98.8% (*n* = 81/82) had medications that were mismatched in their profile. From the 1,207 medications documented across the MyHR and BPMHs, the number of medications documented with either a complete match or a partial match was 59.2% (*n* = 714/1,207). The remaining 40.8% (*n* = 493/1,207) of medications were mismatched. There were 0.3% (*n* = 4/1207) of medications that were listed in the PHR, which the pharmacist had omitted from the BPMHs they obtained. Overall, there was a mean number of six medication mismatches per patient. See Table 3 for medication deviation details.

**Table 3.** Medication deviation details.^a^

	Medication			
Medication category	Complete/partial match, *n* = 714 (%)	Mismatch, *n* = 493 (%)	Total, *n* = 1,207 (%)	*p*-Value
Medication Type				
Regular prescription	548 (78.1)	154 (21.9)	702 (58.1)	<0.001
Regular non-prescription	97 (46.9)	183 (88.4)	207 (17.1)	<0.001
“When required” prescription	30 (41.7)	42 (58.3)	72 (6.0)	0.003
“When required” non-prescription	39 (25.5)	114 (74.5)	153 (12.7)	<0.001
Route				
Oral	597 (60.5)	389 (39.5)	986 (81.7)	0.04
Parenteral	42 (62.7)	25 (37.7)	67 (5.6)	0.61
Others	75 (48.7)	79 (51.3)	154 (12.8)	0.006
ATC classification				
Alimentary tract and metabolism	160 (44.6)	199 (55.4)	359 (29.7)	<0.001
Blood and blood forming organs	65 (65.0)	35 (35.0)	100 (8.3)	0.24
Cardiovascular system	203 (77.8)	58 (22.2)	261 (21.6)	<0.001
Dermatologicals, sensory, various	34 (54.8)	43 (69.4)	62 (5.1)	0.008
Genitourinary, sex hormones, systemic hormonal preparations	39 (76.5)	12 (23.5)	51 (4.2)	0.01
Anti-infective and anti-parasitic agents	15 (65.2)	8 (34.8)	23 (1.9)	0.67
Musculoskeletal system and antineoplastics	32 (62.7)	19 (37.3)	51 (4.2)	0.66
Nervous system	127 (58.8)	89 (41.2)	216 (17.9)	0.94
Respiratory system	39 (56.5)	30 (43.5)	69 (5.7)	0.71

*Note*: ATC: Anatomical Therapeutic Chemical.

a The percentages presented in Table 3 were calculated using the following denominators: (Complete/Partial match)/Total; Mismatch/Total; and Total/1,207.

### Secondary objective – Category, type, severity of deviations and associated risk factors

Of the total 1,207 medications, there were 38.1% (*n* = 460/1,207) medications with a complete match; 21.0% (*n* = 254/1,207) with a partial match; and 40.8% (*n* = 493/1,207) with a mismatch (see Table 2). However, only 10.3% (*n* = 51/493) of the mismatches were deemed a clinically relevant mismatch, and the remaining 89.7% (*n* = 442/493) deemed not clinically relevant. Overall, of the 1,207 medications documented, 4.2% (*n* = 51/1,207) had a clinically relevant deviation.

When examining the mismatches, 71.8% (*n* = 354/493) were drug omissions and 28.2% (*n* = 139/493) drug commissions. The drug omissions were predominantly non-prescription medications (73.4%; *n* = 260/354), and the drug commissions were predominantly prescription medications (73.4%; *n* = 102/139). The remaining 5.7% (*n* = 28/493) were categorised as “other,” and included reasons such as “prescriber intentions unclear,” “patient compliance” and “variable-dosing.”

The multivariable logistic regression showed that a medication was more likely to be mismatched, rather than completely/partially matched, if it was a regular non-prescription medication, “when-required” prescription medication or “when required” non-prescription medication compared to a regular prescription medication. In addition, medications were more likely to have a deviation if they were administered parenterally compared to oral medicines (see Table 4). Whether a medication had a deviation was not significantly related to its ATC classification.

**Table 4.** Multivariable logistic regression for risk factors associated for medications being mismatched.^a^

Variable	Adjusted odds ratio (95% CI)	*p*-Value
Medication type		
Regular prescription	Reference	Reference
Regular non-prescription	7.33 (5.0–10.74)	<0.001
“When-required” prescription	5.83 (3.34–10.17)	<0.001
“When-required” non-prescription	11.27 (7.14–17.79)	<0.001
Medication route		
Oral	Reference	Reference
Parenteral	2.21 (1.22–4.00)	0.009
Others	1.48 (0.73–3.00)	0.28
ATC classification		
Cardiovascular	Reference	Reference
Alimentary tract and metabolism	1.08 (0.69–1.70)	0.74
Blood and blood forming organs	0.73 (0.40–1.32)	0.29
Dermatologicals, sensory, various	0.88 (0.37–2.13)	0.78
Genitourinary, sex hormones, systemic hormonal preparations	0.99 (0.47–2.07)	0.98
Anti-infective and anti-parasitic agents	1.67 (0.66–4.27)	0.28
Musculoskeletal system and antineoplastics	1.19 (0.58–2.45)	0.64
Nervous system	0.81 (0.50–1.31)	0.39
Respiratory system	0.72 (0.30–1.71)	0.46

*Note*: ATC: Anatomical Therapeutic Chemical.

a The dependent variables were chosen as a reference due to their frequent usage. However, this does not imply that no mismatches occurred in these categories.

## Discussion

The focus of this study was the extent of concordance between MyHR and the pharmacist-obtained BPMH. There were 59.2% of documented medications with a complete or partial match between MyHR and the pharmacist-obtained BPMH; and a mean of six mismatches per patient. Although MyHR captures historical medication information and is able to be personally controlled by the health consumer, it is not intended or able to be used as an alternative to a BPMH. This study showed that MyHR offers a valuable adjunct to clinicians creating a BPMH for a health consumer.

This study showed that the MyHR can potentially be used to support the transfer of medication information upon hospital admission as a secondary source. It is important to evaluate the impact of the results on the different healthcare professionals who utilise MyHR differently in practice. In a mixed-methods study exploring the perception of pharmacists and physicians on the use and barriers of MyHR, it was reported that more pharmacists use MyHR compared to physicians (Mullins et al., 2021). Both healthcare professionals reported that MyHR allowed more efficient patient care, especially after hours when other sources are unavailable, such as a patient’s community pharmacy. However, a distinct difference between the pharmacists and physicians was the level of trust in the information in MyHR. While pharmacist’s reported trust concerns associated with outdated or irrelevant information, and therefore tended to use MyHR as an additional, or secondary source, physicians used MyHR to replace other sources of information and deemed MyHR as valid and trustworthy (Mullins et al., 2021).

From the total 1,207 medications documented from the BPMHs and MyHRs, 38.1% had a complete match, 21.0% had a partial match, and 40.8% were mismatched. In the context of a physician’s workflow and their trust in the medication profile in MyHR, combining complete and partial match may potentially be over-stating the accuracy of PHRs. Physicians would be advised to exercise caution when using MyHR as a replacement source of information. However, based on a pharmacist’s workflow, combining complete and partial match would be acceptable, given that MyHR is not the primary or sole source of information and having the medications listed in the MyHR is a sufficient prompt for pharmacists when obtaining a BPMH for patients upon hospital admission.

Our study found higher rates of medication mismatches than the study conducted by van der Nat et al. (2021) in the Netherlands, which reported 17.9% medication mismatches when patients were prompted to actively engage with their medication profile. In the Netherlands, a patient’s medication history is obtained using a nationwide electronic system, the *Medication Record System*, which is based on the dispensing data of all pharmacies. In the van der Nat et al. (2021) study, the patient’s medication list in the patient’s PHR was compared to the pharmacist-documented BPMH and found that patients were able to accurately record their medications, with 82.1% of medication information being correct. However, their study involved prompting the patient to verify the medications in their PHR by either confirming, modifying, ceasing or adding medications 2 weeks prior to admission, which does not align with current practices in Australia. It is noteworthy that the cohort of patients in the study by van der Nat et al. (2021) were elective admissions to hospital and were prompted 2 weeks prior to admission, which implies that this cohort of patients was healthier and this could further account for the greater rate of accuracy (van der Nat et al., 2021). Of all medications in the current study with a mismatch deviation, 10.3% had potentially clinically relevant implications. This is similar to the study by van der Nat et al. (2021), which reported that 7.5% (*n* = 24/334) of medication deviations from the national PHR were clinically relevant (i.e. may cause patient harm) (van der Nat et al., 2021). This highlights that although patient prompting increases overall accuracy, the proportion of clinically relevant deviations remains similar.

Although few medication deviations were clinically relevant in our study, these were mainly due to drug commissions. Medication commissions that were present in the patients’ PHR were predominantly prescription medications, which primarily occurred due to automatic-uploads of connected healthcare systems. Prescription medication commissions require a manual update by either the primary care physician, community pharmacist or patient once they have been ceased. Our study found that commissions were predominantly prescription medications from “alimentary tract and metabolism” and “nervous system” category. However, van der Nat et al. (2021) reported that these prescription medications were predominantly from the “anti-infectives for systemic use” category. This difference may have occurred because our study excluded medications that were short-course or had a defined duration, such as those from the “anti-infectives for systemic use” category. Consequently, prescription medications from these categories, or those with a defined duration, should be screened with caution upon hospital admission, as the process to communicate medication discontinuation is inefficient (Linsky and Simon, 2013; Uitvlugt et al., 2019; van der Nat et al., 2021).

The majority of medication deviations found in our study were considered not-clinically relevant. Such deviations were primarily due to drug omissions from over-the-counter medications. This required manual entry into the patient’s PHR by the patient or their community pharmacy; or to be captured within a hospital discharge summary or GP’s shared health summary. Similarly, a study conducted by Uitvlugt et al. (2019) in the Netherlands, which examined the validity of the medication dispensing records in the Nationwide Medication Record System, reported that omissions were predominantly over-the-counter medications. However, the Uitvlugt et al. (2019) study evaluated the accuracy and completeness of the Netherlands’ nationwide EHR compared to a pharmacy technician’s BPMH for patients upon hospital admission without patient prompting (Uitvlugt et al., 2019). In the Netherlands, patients must consent to each pharmacy for the exchange of information between different pharmacies in their EHR (Uitvlugt et al., 2019). The study reported that the EHR captured 67% (*n* = 322/478) of medications (Uitvlugt et al., 2019). However, their national EHR auto-populated information from dispensing data and did not capture non-dispensed medications (Uitvlugt et al., 2019). In comparison, the van der Nat et al. (2021) reported less over-the-counter related medication deviations, as a result of prompting patients to update their PHR, thereby increasing completeness to 82.1% (*n* = 1441/1756). Contrastingly, Uitvlugt et al. (2019) did not prompt patients to engage and update their medication list in their PHR prior to admission, which aligns with real-time practices in Australia. However, it is advantageous that the Australian national PHR allows GPs to upload shared health summaries, hospitals to upload discharge summaries that do contain non-dispensed medications, and patients to enter their own medications.

Our study also showed that the number of medication deviations in the PHR is affected by the time-frame assessed by healthcare professionals to obtain a medication history. Our study assessed chronic medications (i.e. medications for chronic conditions) as active if they had been listed in the PHR within six months of admission and reported 40.8% (*n* = 493/1,207) mismatched medications, with commissions being the most common deviation type. The study by van der Nat et al. (2021) assessed all dispensed medications in all the past years since inception, and reported that the most common medication deviation for EHRs was commissions of prescription medications (van der Nat et al., 2021). In comparison, Uitvlugt et al. (2019) assessed chronic medications and considered them active if they had been dispensed within one month of admission. Consequently, their study reported a notably lower proportion of mismatched medications, 23.6% (*n* = 113/478), with omissions being the most common deviation type (Uitvlugt et al., 2019). This raises the question as to the time frame healthcare professional should assess when using PHRs to obtain a BPMH. Furthermore, from 1 September 2023, the Australian Government programme that subsidises medications has regulated that patients with chronic conditions are able to receive a 60-day supply of their medication for the price of a single prescription, rather than the previous 30-day supply (Australian Government Department of Health and Aged Care, 4 July 2023). This may mean that healthcare providers may need to consider a longer time frame for patients with chronic conditions when using PHRs to obtain a BPMH upon hospital admission.

A recently published Australian study evaluated the accuracy of a cloud-based repository of prescription and dispensing records, *MedView*, which functions similarly to MyHR (Elliott et al., 2023). Although MedView and MyHR are both cloud-based repositories of prescribed and dispensed medications, MyHR uniquely allows information flow from various connected systems, as well as allowing consumers to engage and exercise various security controls. Elliott et al. (2023) also concluded that MedView data from cloud-based repositories need to be verified with additional sources including the patient and/or their carer. The uniqueness of MyHR was reaffirmed by a study that compared the EHRs between 11 countries and reported that 18% (*n* = 2/11) of countries allowed a level of editing by consumers, with Australia being one of them (Nohr et al., 2016). Overall, there is limited literature that explores the concordance between medication lists in EHRs and BPMHs obtained by pharmacists.

Future studies could evaluate the potential utility of PHRs, by comparing the accuracy of BPMHs obtained with and without a PHR. Other future studies or initiatives may consider changes in the system’s design that targets the patient’s primary physician to action certain trigger prompts. An example of an actionable item triggered by the system for the physician to address might include actively ceasing a medication that may not have been prescribed for a certain duration or might be a duplication in therapy. Another potential change could be the involvement of community pharmacists actively uploading over-the-counter medications into the PHR. Based on the results of this study, different stakeholders could be engaged to improve the accuracy of PHRs.

Overall, PHRs could be improved by promoting greater awareness and thereby encouraging key stakeholders, such as patients, to engage with their MyHR, as well as healthcare providers and their organisations to securely connect their clinical information systems to MyHR to enable medication information to flow. Additionally, irrespective of whether a healthcare setting is private or public, either can register for a unique HPI-O number and connect to MyHR. By maximising the number and type of healthcare settings and providers that are connected and accessing PHRs, this can further enhance the concordance of PHRs with pharmacist-obtained BPMHs.

### Limitations

First, it was not possible to blind the pharmacist obtaining the BPMHs, and therefore the Hawthorne effect would have occurred to an extent. Second, only one investigator completed the data, and due to time restrictions, a convenience sample was used, which may limit generalisability. The convenience sample was based on the normal workflow of the pharmacist, which involves prioritising high-risk patients. Consequently, there was no randomisation, and this implies a degree of selection bias. Third, the medication deviations were categorised based on the clinical judgement of a panel of clinicians, in which there may be a level of subjectiveness. Fourth, the study assessed medications from a public-healthcare perspective and therefore results should be carefully applied in a predominantly private-healthcare setting, such as that in the United States, as patient engagement might be influenced by consequential healthcare insurance implications.

## Conclusion

In conclusion, despite PHRs being able to capture medications that are clinically relevant, it is important to note that PHRs will not replace, but rather complement the process of interviewing patients to obtain a BPMH as a secondary source (Linsky and Simon, 2013; Russell et al., 2013), and should therefore, be routinely considered by pharmacists as part of their BPMH process. The Australian national PHR, MyHR, may be a secondary source to either confirm a patient’s prescription medications or be used as a starting point for a BPMH for patients upon hospital admission where no other source is available or the patient is unable to communicate.
